# Occurrences of Threatened Species included in the Third Edition of the Red Data Book of the Komi Republic (Russia)

**DOI:** 10.3897/BDJ.9.e73763

**Published:** 2021-10-21

**Authors:** Svetlana Degteva, Anatoly Bobretsov, Yury Bobrov, Modest Dolgin, Mikhail Dulin, Nickolay Filippov, Nadezhda Goncharova, Janolof Hermansson, Vladimir Kanev, Dmitry Kirillov, Irina Kirillova, Olga Kirsanova, Sergey Kochanov, Alla Kolesnikova, Tatyana Konakova, Andrey Korolev, Denis Kosolapov, Oksana Kulakova, Ekaterina Kulyugina, Olga Loskutova, Elena Melekhina, Oleg Mineev, Yuri Mineev, Vladimir Morozov, Gleb Nakul, Marina Palamarchuk, Elena Patova, Sergej Pestov, Anatoly Petrov, Irina Poletaeva, Vasily Ponomarev, Tatiana Pystina, Yury Rebriev, Roman Romanov, Natalya Selivanova, Anton Shiryaev, Tatyana Shubina, Irina Sterlyagova, Andrey Tatarinov, Boris Teteryuk, Ludmila Teteryuk, Zinaida Ulle, Olga Valuyskikh, Alexander Zakharov, Galina Zheleznova, Aurika Zinovyeva, Yuriy Dubrovskiy, Boris Gruzdev, Anna Ichetkina, Vera Martynenko, Nadezhda Oplesnina, Vera Panova, Irina Romanova, Mikhail Rubtsov, Leonid Rybin, Nataliya Semenova

**Affiliations:** 1 Institute of Biology of Komi Scientific Centre of the Ural Branch of the Russian Academy of Sciences, Syktyvkar, Russia Institute of Biology of Komi Scientific Centre of the Ural Branch of the Russian Academy of Sciences Syktyvkar Russia; 2 Pechoro-Ilych State Nature Reserve, Yaksha, Russia Pechoro-Ilych State Nature Reserve Yaksha Russia; 3 Pitirim Sorokin Syktyvkar State University, Syktyvkar, Russia Pitirim Sorokin Syktyvkar State University Syktyvkar Russia; 4 Department of Physical Planning, Ludvika, Sweden Department of Physical Planning Ludvika Sweden; 5 All-Russian Research Institute for Environment, Moscow, Russia All-Russian Research Institute for Environment Moscow Russia; 6 Southern Scientific Centre of Russian Academy of Sciences, Rostov-on-Don, Russia Southern Scientific Centre of Russian Academy of Sciences Rostov-on-Don Russia; 7 Komarov Botanical Institute of the Russian Academy of Sciences, Saint Petersburg, Russia Komarov Botanical Institute of the Russian Academy of Sciences Saint Petersburg Russia; 8 Institute of Plant & Animal Ecology (IPAE) Ural Branch of the Russian Academy of Sciences (UrB RAS), Ekaterinburg, Russia Institute of Plant & Animal Ecology (IPAE) Ural Branch of the Russian Academy of Sciences (UrB RAS) Ekaterinburg Russia

**Keywords:** regional Red Data Book, Komi Republic, European north-east Russia, Urals, conservation, rare species, GBIF

## Abstract

**Background:**

The purpose of the data paper was to introduce into scientific literature the results of scientific work carried out for the third edition of the 'Red Data Book of the Komi Republic'. The article reflects methodological approaches to the formation of a list of rare and in need of protection species and describes the corresponding datasets published in GBIF.

**New information:**

Information about 7,187 occurrences of 438 rare species and infraspecies included in the third edition of the 'Red Data Book of the Komi Republic' have been published.

## Introduction

The diversity of species, amongst microscopic organisms, plants, animals and fungi that were formed on Earth after a long-term evolution process, constitutes the basis of the biosphere and individual ecosystems that make up this Planet. The development of human societies is associated with an ever-increasing impact on nature; in particular, with a decrease in the number of living biological species due to changes in their natural habitats and, in some cases, to direct elimination. As natural communities become less diverse, their resistance to anthropogenic impact and resilience decrease (Loreau and de Mazancourt 2013, Oliver et al. 2015, Leitão et al. 2016). This has demonstrated that a rational nature management is needed and that the conservation of biological diversity should be a priority.

Special attention is needed for those species of animals, plants and fungi whose populations, due to natural reasons or human activity, reduce their numbers and become less common. Since the second half of the 20^th^ century, one of the most important mechanisms contributing to their preservation has been the creation of Red Data Books at the international, regional and local levels (Rodrigues et al. 2006).

In accordance with the Federal Law № 7-FZ of 10 January 2002 "On Environmental Protection" (Федеральный закон "Об охране окружающей среды" от 10.01.2002 N 7-ФЗ), the creation of the Red Data Book of the Russian Federation and the Red Data Books of the regions of the Russian Federation has been established to protect rare and endangered species of animals, plants and fungi, monitor their state and develop and implement measures aimed at preserving and restoring the number of their populations (Popov et al. 2017).

In the Komi Republic, work aimed at preserving rare species has been carried out since the 1980s. In 1982, the monograph 'Rare and in need of protection animals and plants of the Komi ASSR' was published (Roshchevskij 1982); it summarises all the information available at that time about rare species of the flora and fauna of the region. Based on the scientific argumentation prepared by specialists of the Institute of Biology of the Komi branch of the USSR Academy of Sciences (Syktyvkar), the list of rare plant species of the Komi Republic was approved by Resolution № 82 of the Council of Ministers of the Komi ASSR of 24 February 1983 (Постановление Совета Министров Коми АССР от 24 февраля 1983 г. N 24).

In 1995, the Ministry of Natural Resources and Environmental Protection initiated the publication of the regional Red Data Book. At the initial stage, the specialists of the Institute of Biology of the Komi Scientific Center of the Ural Branch of the Russian Academy of Sciences updated the lists of rare and endangered fungi, flora and fauna species proposed for inclusion in the new edition of the regional Red Book and clarified the categories of rarity status for each one of them.

When compiling the lists, the criteria for identifying rare and endangered animal species were in accordance with the 'Strategy for Preserving Rare and Endangered Species of Animals, Plants and Fungi', approved by Order of the Ministry of Natural Resources of the Russian Federation on 6 April 2004 N 323 (Приказ Министерства природных ресурсов Российской Федерации от 6 апреля 2004 года N 323 "Об утверждении Стратегии сохранения редких и находящихся под угрозой исчезновения видов животных, растений и грибов"). Amongst naturally rare species, considered potentially vulnerable due to their biological features, were taxa that met one or more of the following criteria:

- low population size,
- small range area (relic, endemic or range border),
- low density,
- low ecological valence (stenobionticity or high specialisation),
- low rate of population reproduction,
- negative response to human presence.

In addition, the following limiting factors and threats were taken into account when including species in the Red Lists: 1) disturbances and/or pollution of soil and vegetation in the territory of a species (such as exploration, extraction and transportation of hydrocarbons, mining of solid minerals, quarrying, laying communications and power lines, construction, passage of tracked vehicles in a snowless period, sleigh driving and overgrazing by reindeers, grazing, ploughing, land reclamation, swamp drainage, peat extraction, felling of forest/shrubs and trees and haymaking), 2) atmospheric pollution (due to combustion of hydrocarbons, transboundary transport, emissions from industrial enterprises and transport, for example, 3) fires, burning dry grass and felling residues, 4) unregulated tourism and recreation, 5) collection of plants (or their parts) for bouquets, 6) procurement of plants (or their parts) as medicinal raw materials or for use as food, 7) digging out plants (or their parts) for transplanting into gardens and household plots (or for the purpose of introduction), 8) violation of forest litter regulations, 9) violation of the hydrological regime of water bodies and watercourses and technogenic pollution of water bodies, 10) pollution of groundwater, 11) reduction of flow of rivers, 12) regulation of river flow (creation of reservoirs), 13) poaching, 14) accidental shooting, 15) destruction of nests, 16) holes, 17) destruction of forage lands, 18) collection by collectors, 19) reduction in the number of spawning grounds and breeding places, 20) causing stress during the breeding season, 21) direct destruction by humans, 22) death in fishing nets or traps on the roads and 23) overgrowing of meadows and fields with shrubs and trees.

The publication of the Red Data Book of the Komi Republic was established by Decree № 175 of the Head of the Komi Republic, from 18 May 1998 (Указ Главы Республики Коми от 18.05.1998 № 175 'Об учреждении Красной книги Республики Коми'); the book has been published three times (Taskaev 1998, Taskaev 2009, Degteva 2019). The Red Data Book of the Komi Republic is the regional legal mechanism for the conservation and restoration of rare and endangered species of animals, plants and fungi and their population diversity to ensure their sustainable existence. Its first official publication included 110 species of fungi (including lichens), 394 species of plants and 107 species of animals; a total of 611 taxa.

For determining the correct protection status of each species (and subspecies) listed in the first edition of the Red Data Book of Komi Republic (Taskaev 1998), they were assigned to one of the categories of rarity status (0-4) adopted in the 'Red List of the International Union for Conservation of Nature' (IUCN) (Lucas et al. 1978). In addition to these, the first edition of the 'Red Data Book of the Komi Republic' (Taskaev 1998) introduced category 5. The criteria used for assigning the rarity status categories to plants, fungi and animals differed. The Book included plants and fungi that were quite common, but whose abundance might decrease due to anthropogenic impact and thus required biological supervision. For the kingdom Animalia, species that had been restored or were restoring their numbers were included in category 5. Hunting, fishing and collecting animals, plants and fungi belonging to the species listed in the regional Red Data Book were prohibited throughout the territory of the Republic, except in cases provided for by the legislation of the Russian Federation and the Komi Republic.

According to the Decree of the Head of the Komi Republic № 175, dated 18 May 1998, 'On the establishment of the Red Data Book of the Komi Republic' (Указ Главы Республики Коми от 18.05.1998 № 175 'Об учреждении Красной книги Республики Коми'), the Book should be published once every 10 years. In the periods between reissuing the Book, a targeted collection, processing, systematisation and analysis of data on the biology and abundance of the species that should be protected in the region should be carried out, together with the identification of those limiting factors and threats to the stable state of their populations, in order to take the necessary measures for effective protection of such species. These tasks should be carried out by specialists from scientific and environmental organisations and higher education institutions by order of the Ministry of Natural Resources and Environmental Protection of the Komi Republic, which is the institution responsible for updating the Red Data Book.

Through Order № 79 of the Ministry of Natural Resources and Environmental Protection of the Komi Republic, dated 12 February 2008 (Приказ министерства природных ресурсов и охраны окружающей среды Республики Коми от 12 февраля 2008 года №79 'Об утверждении перечней (списков) объектов растительного и животного мира, занесенных в Красную книгу Республики Коми'), a new list of rare fauna and flora species including fungi was approved. The second edition of the regional Red Data Book was published in 2009 (Taskaev 2009). When preparing it, the compilers were guided by the 'Methodological Recommendations for maintaining the Red Data Book of the regions of the Russian Federation' (letter № 02-12-53/5987 of the Ministry of Natural Resources of the Russian Federation dated 27 July 2006 (Письмо Министерства природных ресурсов Российской Федерации от 27 июля 2006 года № 02-12-53/5987). The second edition of the 'Red Data Book of the Komi Republic' (Taskaev 2009) had a general structure similar to that of the 'Red Data Book of the Russian Federation' (Danilov-Danilyan 2001, Bardunov and Novikov 2008). In particular, the same criteria and categories for determining the rarity status of species were used:

- 0 – Probably extinct. Species (subspecies or populations) previously known to inhabit the territory of Komi Republic whose presence in nature has not been recorded after continuous surveying efforts (for invertebrates, the last 100 years and for vertebrates, the last 50 years).
- 1 – Endangered species. Species (subspecies or populations) whose number of individuals has decreased to a critical level or whose habitats have decreased so much in number that they may disappear in the near future.
- 2 – Vulnerable species. Species (subspecies or populations) with a steadily decreasing number and/or distribution areas, which, under the further influence of factors that reduce their number, may, in a short time, fall into the category of endangered.
- 3 – Rare species. Species (subspecies or populations) with naturally low abundance, distributed in a limited territory (or water area) or sporadically occurring in significant territories (or water areas) that need special protection measures to ensure their survival.
- 4 – Indeterminate by status species. Species (subspecies or populations) that probably belong to one of the previous categories, but whose current state in nature is not well documented or that do not fully meet the criteria of all other categories, but need special protection measures.
- 5 – Restored and recovering species. Species (subspecies or populations) whose abundance and distribution, either due to the influence of natural causes or adopted protection measures, have begun to recover and are approaching a state where they will not need any special protection measures.

The second edition of the 'Red Data Book of the Komi Republic' (Taskaev 2009) included 124 species of fungi (including lichens), 311 species of plants and 99 species of animals, a total of 534 taxa. The publication included 35 species listed in the 'Red Data Book of the Russian Federation' (Danilov-Danilyan 2001, Bardunov and Novikov 2008) whose categories also defined their protection at the federal level.

In the 10 years that have passed since the publication of the second edition of the 'Red Data Book of the Komi Republic' (Taskaev 2009), specialists have received new information about the diversity of the flora, fauna, lichen biota and mycobiota of the region. New taxa and habitats of rare species have been identified. During all this time, scientists have conducted systematic inventories of members of the local fauna and flora complexes for the timely identification of species at high risk of loss.

Special attention was paid to the inventory of objects of the nature protected fund and on the territories of local populations of many species listed in the Red Data Books of both the Russian Federation and the Komi Republic. It has been confirmed that populations of many rare species are located within Nature Protected Areas (NPAs). A key role in the preservation of habitats and populations of rare species of plants, animals and fungi is played by NPAs of federal significance, such as the Pechora-Ilych Reserve and the Yugyd Va National Park (Degteva and Ponomarev 2014). These data, as well as information from literature sources and materials stored in herbariums and museum collections in Moscow, St. Petersburg, Yekaterinburg, Perm and Syktyvkar, have been considered when compiling the updated lists of animals, plants and fungi included in the third edition of the 'Red Data Book of the Komi Republic' (Degteva 2019). These lists include 532 taxa: 150 fungi (including lichens), 314 plants (including *Nostocpruniforme* C. Agardh ex Bornet et Flahault, which actually belongs to the Bacteria Kingdom) and 68 animals. The categories of the rarity status of each species were assigned according to the system adopted in the 'Red Book of the Russian Federation' (Danilov-Danilyan 2001, Bardunov and Novikov 2008) and the second edition of the 'Red Data Book of the Komi Republic' (Taskaev 2009).

The Komi lists include 47 species rare plants, animals and fungi listed in the 'Red Data Book of the Russian Federation' (Danilov-Danilyan 2001, Bardunov and Novikov 2008) (Table 1).

Most of the taxa listed in the 'Red Data Book of the Russian Federation' (Danilov-Danilyan 2001, Bardunov and Novikov 2008) have been included in the third edition of the 'Red Data Book of the Komi Republic' (Degteva 2019) with the same category of rarity status. The exceptions are *Menegazziaterebrata* and *Huchotaimen*, which are probably extinct in the region, *Lobariapulmonaria*, *Stereocaulondactylophyllum* and *Cottusgobio*, *Circusmacrourus*, *Cygnusbewickii* and *Gaviaarcticaarctica* whose populations are quite numerous in the territory of the Komi Republic. Besides, this regional Red Data Book includes eight taxa whose populations need special attention due to the current conditions of their natural habitats and that are included in the Annex to the 'Red Data Book of the Russian Federation' (Danilov-Danilyan 2001, Bardunov and Novikov 2008). It is noteworthy that one species of vascular plants, two species of lichens and 42 animal species from the Komi lists are also included in the IUCN Red List of Endangered Species (IUCN 2021).

Before the publication of this paper, the 42 datasets with occurrences of rare species have been published in the GBIF: Adojaan et al. (2019), Auer et al. (2021), Beshkarev et al. (2019), Bobretsov (2019), Brianskaia et al. (2021), Community of Cris and Melechin (2019), De Vos and Creuwels (2021), Degteva et al. (2021b), Doubt (2021), Erokhin and Vorobiev (2019), Filippova et al. (2020), Hagemeyer and Blair (2017), European Nucleotide Archive (EMBL-EBI) (2019), Johannessen and Johnsen (2021), Karyakin et al. (2020), Kirillov and Kirillova (2019b), Kirillov and Kirillova (2019a), Kirillov and Kirillova (2020), Konakova (2020), Konakova and Kolesnikova (2021), Konakova et al. (2021), Kovtonyuk et al. (2021), Lahti (2017), Lomonosov Moscow State University (2018), MNHN (2021), Palamarchuk and Kirillov (2019), Pärtel (2021), Petrosyan (2019), Consortium (2016), Rebriev (2021), Norwegian University of Science and Technology (2021), Reshetnikova et al. (2020), Seregin (2021), Seregin and Stepanova (2021), Shashkov (2019), Soudzilovskaia et al. (2021), Ueda (2021), UiT The Arctic University of Norway (2021) (2021), Ukolov et al. (2019), Vaganov and Maratkanova (2021), Zheleznova et al. (2020a), Zheleznova et al. (2020b). The query result containing information on occurrences of rare species within the administrative boundary of the Komi Republic, published in GBIF, can be reached at https://api.gbif.org/v1/occurrence/download/request/0328832-200613084148143.zip. This query result was obtained with the rgbif (Chamberlain and Boettiger 2017) package of R programming language (R Core Team 2021).

The following datasets contain valuable information about rare taxa finds: Seregin (2021) - 66 spp., Zheleznova et al. (2020a), Zheleznova et al. (2020b) - 52 spp., Ueda (2021) - 30 spp., Hagemeyer and Blair (2017) - 23 spp. Amongst these, data on more than 1200 occurrences were published by specialists of the Institute of Biology of the Komi Science Center of the Ural Branch of the Russian Academy of Sciences: Kirillov and Kirillova (2019a), Kirillov and Kirillova (2019b), Kirillov and Kirillova (2020) - 658 occurrences; Konakova (2020), Konakova and Kolesnikova (2021) - 325 occurrences; Zheleznova et al. (2020a), Zheleznova et al. (2020b) - 284 occurrences; Konakova et al. (2021) - 15 occurrences and Palamarchuk and Kirillov (2019) - 15 occurrences. Information about a significant number of rare taxa finds is also given in the articles from Seregin (2021) with 215 occurrences, Hagemeyer and Blair (2017) - 194 occurrences, Beshkarev et al. (2019) - 165 occurrences, Brianskaia et al. (2021) - 82 occurrences, and Ueda (2021) - 36 occurrences. Our publication offers information about the largest number of rare species identified until now (n = 470) and the largest number of occurrences (7186).

## General description

### Purpose

The data paper was created according to the concept described in works of Vishwas Chavanand and Lyubomir Penev (Chavan and Penev 2011, Penev et al. 2017) for description of two original datasets (Degteva et al. 2021a, Degteva et al. 2021b) concerning the third edition of Red Data Book of Komi Republic (Degteva 2019).

## Project description

### Title

Occurrences of Threatened Species included in the Third Edition of the Red Data Book of the Komi Republic (Russia)

### Personnel

S. Degteva, A. Bobretsov, Y. Bobrov, M. Dolgin, M. Dulin, N. Filippov, N. Goncharova, J. Hermansson, V. Kanev, D. Kirillov, I. Kirillova, O. Kirsanova, S. Kochanov, A. Kolesnikova, T. Konakova, A. Korolev, D. Kosolapov, O. Kulakova, E. Kulyugina, O. Loskutova, E. Melekhina, O. Mineev, Yu. Mineev, V. Morozov, G. Nakul, M. Palamarchuk, E. Patova, S. Pestov, A. Petrov, I. Poletaeva, V. Ponomarev, T. Pystina, Y. Rebriev, R. Romanov, N. Selivanova, A. Shiryaev, T. Shubina, I. Sterlyagova, A. Tatarinov, B. Teteryuk, L. Teteryuk, Z. Ulle, O. Valuyskikh, A. Zakharov, G. Zheleznova, A. Zinovyeva, Y. Dubrovskiy, B. Gruzdev, A. Ichetkina, V. Martynenko, N. Oplesnina, V. Panova, I. Romanova, M. Rubtsov, L. Rybin, N. Semenova

### Study area description

Populations of rare species were examined in the territories of the Yugyd va National Park, the Pechora-Ilych State Natural Biosphere Reserve and 13 NPAs of the Komi Republic. It was found that, in most of the NPAs, there are no threats to the existence of populations of rare species. Based on the analysis of available information and field research data (2016-2018), the authors compiled updated lists of 548 rare species of flora and fauna, proposed for inclusion in the new edition of the Red Data Book of the Komi Republic. Amongst them are 65 species of real mushrooms, 88 species of lichens, 10 species of algae (including *Nostocpruniforme*), 71 species of bryophytes, 234 species of vascular plants, 43 species of invertebrates, six species of fish, one species of amphibians, 26 species of birds and four species of mammals. According to the research results of 2009-2018, it was proposed to exclude 75 taxa from the Red Data Book of the Komi Republic. Amongst them there are seven species of fungi, one lichen, one species of algae, 14 species of bryophytes, 19 species of vascular plants, 22 species of invertebrates, one species of amphibians, eight species of birds and one species of mammals. Most of them were included in Appendix 1 to the regional Red Data Book as taxa that need constant control of the number of populations in nature.

### Design description

An inventory of information on the distribution, habitats, number and state of populations of rare species of plants, animals and fungi obtained for the period from 2009 to 2015 was carried out. The lists of species for which it is necessary to conduct additional research have been clarified. A survey of forestry and hunting experts and the population of the Komi Republic was carried out in order to identify the habitats of birds of prey and anseriformes and mammals included in the 'Red Data Book of the Komi Republic' (Degteva 2019). The revision of the SYKO herbarium collections was carried out. Field studies were carried out, aimed at identifying key habitats, assessing the abundance of populations of plants, animals and fungi species listed in the 'Red Data Book of the Komi Republic' (Taskaev 2009), for which data gaps were identified. Photographing of rare species and their habitats was carried out. The lists and categories of the rarity status of species proposed for inclusion in the third edition of the Red Data Book of the Komi Republic have been clarified. The updated maps of the distribution of rare species have been compiled. Supplements to the essays on rare species have been prepared for the new edition of the Red Data Book of the Komi Republic. Methods have been prepared for calculating the amount of harm caused to objects of the animal and plant world listed in the Red Data Book of the Komi Republic and their habitat and, finally, proposals have been made on the standards for the cost of rare species.

### Funding

The research was supported by the funds of the budget of the Komi Republic allocated for environmental protection. Sponsorship in the organisation of the expedition work was provided by Mondi Syktyvkar JSC and PJSC Gazprom ('Gazprom Transgaz Ukhta'). This work was partly supported by: grant of the Russian Foundation for Basic Research (RFBR) Project Number: 16-44-110167; Projects from the State Tasks of the Institute of Biology of Komi Scientific Centre of the Ural Branch of the Russian Academy of Sciences: № АААА-А19-119011790022-1, № АААА-А17-117112850235-2; and Project of State Tasks of the Komarov Botanical Institute of the Russian Academy of Sciences № 121021600184-6.

## Sampling methods

### Study extent

The dataset contains the occurrences of the species included in the 'Red Data Book of the Komi Republic' (Degteva 2019) and collected within the limits of the Komi Republic. The assessment of the species compliance with the criteria of natural rarity and population or range decline as a result of human impact was made, based on data available in the scientific literature and data stored in the collection funds of herbaria and museums, and on the results of scientific research on biological diversity. In addition, known populations of rare species in the territory of the Komi Republic were monitored between 2009 and 2018 and information was received from local people when performing the surveys and through media materials. When classifying the rarity of a species, we used the criteria and categories of status adopted in the 'Red Data Book of the Russian Federation' (Bardunov and Novikov 2008, Danilov-Danilyan 2001).

### Sampling description

Information about the habitats of rare species was collected during field research. Particular attention was paid to the inventory of NPAs and in the territories within which local populations of many species were concentrated and that were listed in the Red Data Books of the Russian Federation and the Komi Republic (Degteva and Ponomarev 2014). Field studies were carried out mainly by the route method. The method of winter route counts was also used for vertebrates. The data were recorded in field journals together with geobotanical descriptions. For each occurrence, the coordinates were fixed using GPS navigators. All findings of rare species of plants and fungi were confirmed by herbarium collections, that were mainly stored in the herbarium (SYKO) of the Institute of Biology of the Komi Scientific Center of the Ural Branch of the Russian Academy of Sciences. Findings of invertebrates were included in the collections of the scientific museum of the Komi Scientific Center of the Ural Branch of the Russian Academy of Sciences. *Pontastacusleptodactylus* Eschsch, 1823 was recorded in 2017 by local people in the River Serdyel (Nikolaeva 2017, a mass media publication). To obtain additional information about rare bird species, surveys amongst local populations were carried out. Aerial surveys were used to monitor reindeer (*Rangifertarandus* Linnaeus, 1758) populations. To clarify the information on the distribution of rare species, we used the collections of the Herbaria of the Komarov Botanical Institute RAS (LE), the Institute of Plant and Animal Ecology of the Ural Branch of the Russian Academy of Sciences (SVER), the Central Siberian Botanical Garden SB RAS (NS), the Papanin Institute of Biology of Inland Waters (IBWI), the Lomonosov Moscow State University (MW), the Perm State National Research University (PERM), the Pechoro-Ilychskiy State Nature Reserve (PIR), the Botanical Museum of Uppsala University in Sweden (UPS), the Herbarium of Saint Petersburg University (LECB), the Herbarium of Institute of Agricultural and Environmental Sciences of the Estonian University of Life Sciences (TAAM) and the private collection of J. Hermansson, as well as the zoological collections stored in the Museum of the Pitirim Sorokin Syktyvkar State University.

### Quality control

Species identification was made or checked by specialists on the corresponding taxon. The identification of 4215 occurrences (47% of the dataset) of 359 species (76% of the species included in the dataset) was supported by preserved specimens. The taxa names were normalised with help of the GBIF species matching tool (https://www.gbif.org/tools/species-lookup). The dataset was checked for errors and cleaned by methods described by R. Mesibov in A Data Cleaner's Cookbook (Mesibov 2021). The geo-referencing was checked by overlaying the occurrence points and the geographical map from the QGIS Geographic Information System (QGIS Development Team 2021).

### Step description

The authors of this document prepared occurrence data (separate datasets for the taxonomic groups for which they were responsible) of rare species, not previously published in GBIF, with a common xlsx-template, based on the occurrence template recommended by the Integrated Publishing Toolkit (IPT) (https://github.com/gbif/ipt/wiki/occurrenceData#templates). Authors were allowed to add Darwin Core terms as additional fields in their xlsx-files.

The draft checklist of the species included in third edition of Red Data Book of the Komi Republic was prepared with the help of the checklist template recommended by the IPT (https://github.com/gbif/ipt/wiki/occurrenceData#templates). This checklist was matched against the GBIF Backbone checklist using the "Species name matching" tool at gbif.org (https://www.gbif.org/tools/species-lookup) and sent out to the authors of this paper; they checked the information of those taxa for which they were responsible. Authors were allowed to add references in the 'nameAccordingTo' field, especially in those cases in which they were using synonyms or names not listed in the GBIF Backbone. The field 'taxonRemarks' was used to indicate the rarity status of each taxon.

The occurrence datasets prepared by the specialists were merged into a single dataset. The 'taxonID' field was filled with values from the corresponding field from the checklist dataset. All additional fields used by the authors were included in the merged dataset. Most of the values in the fields “decimalLongitude' and 'decimalLatitute' were rounded to two decimal places and the 'coordinateUncertaintyInMeters' field was set to a single value of 3000 (7096 records). The level of georeference data generalising was chosen according to the level of generalising recommended for the most sensitive species in our dataset - the rare bird species. Some records with poor locality data were georeferenced with greater uncertainty of: 5,000 m (79 records), 10,000 m (8 records), 15,000 m (1 record), 25,000 m (2 records), 50,000 m (1 record). Duplicated records, created after the coordinate generalisation, were deleted from the dataset. The records that did not have any information in the 'decimalLongitude', 'decimalLatitute' and 'recordedBy' fields were also deleted from the dataset. Each record in the occurrence dataset also included a URL leading to the corresponding species’ description and images at the Red Data Book of the Komi Republic web page.

## Geographic coverage

### Description

**Figure 1.**
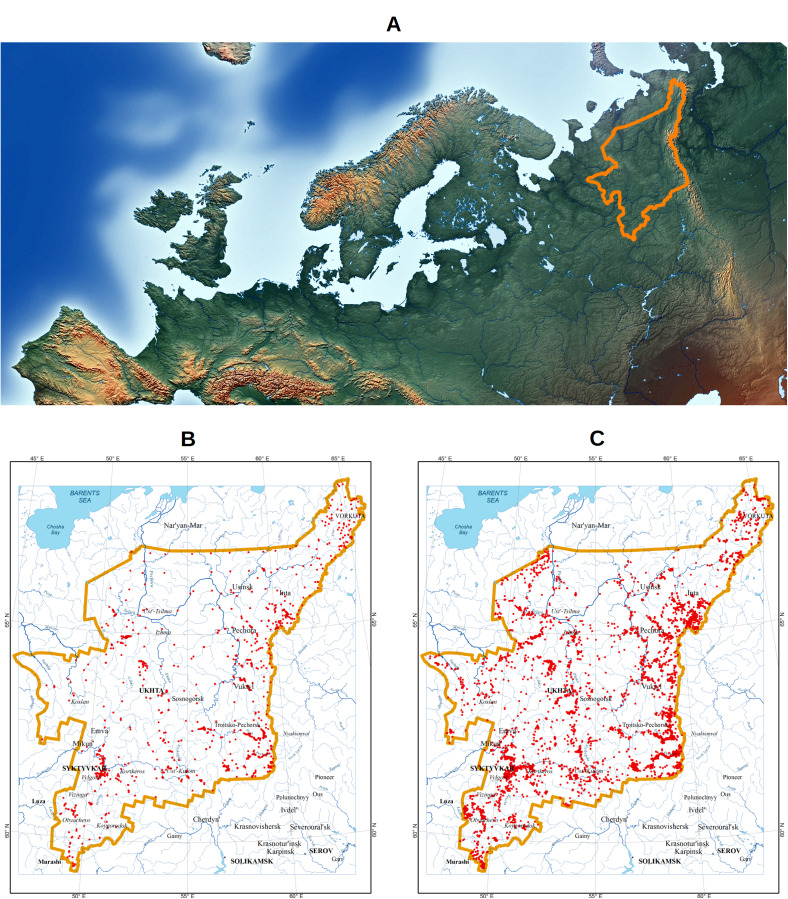
Territory of the Komi Republic and occurrences of species included in the third edition of the 'Red Data Book of the Komi Republic'. **A** The Komi Republic on Europe map (border is highlighted by orange colour); **B** The occurrences (red dots) published in GBIF apart from the dataset described in this data paper; **C** The occurrences (red dots) published in the GBIF dataset (Degteva et al. 2021b) described in this data paper.

The Komi Republic is located north-east of the Russian (East European) Plain and on the western macroslope of the northern part of the Ural Mountains (Fig. 1). The total area of the region is 416 800 km² (https://rkomi.ru/pages/48). The region landscapes are dominated by low-lying areas with elevation markers up to 200 m. Elevated areas of Ural Mountains and Timan Ridge cover about 20% of the Komi Republic. The elevations of the highest mountains of the Northern, Subpolar and Polar Urals are 1617, 1894 and 1500 m, respectively. Excessive humidity and, mainly, flat relief contribute to the development of mires, which occupy about 7.7% of the region's territory (Panev 1964, Degteva and Ponomarev 2014).

The region has severe and continental climate with frequent invasion of arctic air masses from the Arctic Ocean. The climate varies in different parts of the Republic due to its large extension from north to south and from west to east (more than 1000 km in both directions) and to the high variety of relief forms (lowlands, uplands and mountain systems). Most of the Republic is in the Atlantic-Arctic climatic zone with moderate cold (boreal) weather with long winter and short and cool summer seasons. Northwards and in the Urals, the climate becomes arctic and subarctic. The north of the region lies in the permafrost area, where the average annual temperature ranges from –4°С to –6°С, rising to 0–1°С in the south of the Republic. Annual precipitation is not higher than 400–450 mm in the north and 600 mm in the south. In montane areas, it can be up to 1000 mm (Taskaev 1997).

The southern bush tundra, formed by *Betulanana* L., Salix spp. and forest-tundra are dominant vegetation types in the north-east region. Most of the region is covered by taiga, mainly spruce forests dominated by *Piceaobovata* Ledeb. In addition, pine forests (*Pinussylvestris* L.) are widespread throughout the taiga, mainly on sandy terraces along riverbanks and peat soils on the edges of raised bogs. Spruce (*P.obovata*) and fir *(Abies sibirica* Ledeb.) stands and, rarely, cedar pine (*Pinussibirica* Du Tour) forests are spread in the foothills of the Northern Urals. Larch (*Larixsibirica* Ledeb.) stands are common northwards of 64° N and at the Timan Ridge. On the western macroslope of the Urals, along the elevation gradient, vegetation forms several altitudinal belts, including mountain-forest with spruce, fir and cedar pine, spruce, larch and birch forests in the north, subalpine areas where mountain woodlands are combined with bushes, meadows and fragments of mountain tundra and alpine belts.

About 65% of the landscapes of the territory of the Republic are relatively intact. In the foothills and mountains of the Northern and Subpolar Urals within the territory of the Pechora-Ilych Reserve and the Yugyd va National Park, the largest arrays of virgin forests of the European North have been preserved; they have been practically free from any human impact. In 1995, these protected areas were included in the UNESCO World Heritage List as the 'Virgin forests of Komi' (Degteva and Ponomarev 2014). This is the first site in Russia recognised by the UNESCO World Nature Heritage List, which protects 20% of the undisturbed forests of Europe. In total, there are 234 NPAs covering approximately 5.44 million ha (13% of the region area) of the Komi Republic.

NPAs are the key elements of the biodiversity conservation system in the region. Most of the species included in the Red Data Book of the Komi Republic may be found within the limits of NPAs, including 83% of the vertebrates and 80.6% of the invertebrates, 84.1% of the vascular plants, 84.3% of the mosses, 50% of the algae, 94% of lichens and 88% of the fungi (excluding lichens). The state of the rarest plant species populations within NPAs has been assessed as stable.

### Coordinates

59.22 and 68.28 Latitude; 45.87 and 66.07 Longitude.

## Taxonomic coverage

### Description

Together with the GBIF dataset on the occurrences of rare species, a list of all the taxa included in the 'Red Data Book of the Komi Republic' (Degteva 2019) is being published (Table 2). In most cases, the species names coincide with those in the list of species of the GBIF Taxonomic Backbone. The exceptions are two species of Lepidoptera: *Polyommatuserostaimyrensis* Korshunov, 1982 and *Clossianatritonia* (BӧBer, 1812). In some cases, the names of the species used in the Red Data Book are classified as synonyms, but the compilers of the Red Data Book have retained their independent status for environmental protection purposes. This should reduce the risk of negative impact on regional/unique populations whose taxonomic status may be revised or is currently controversial.

The sources listed in the 'nameAccordingTo' field of the checklist dataset contain 17 references (Arbeitsgruppe Characeen Deutschlands Lehrstuhl für Ökologie der Universität 2016, Cherepanov 1995, Eloranta and Kwandrans 2007, Hara 1966, Ignatov et al. 2006, Index Fungorum 2021, Kanyukova 2006, Komárek 2013, Krause 1997, Kuzmin 2012, Lvovskij and Morgun 2007, Söderström et al. 2016, Soó 1969, Stepanyan and Pavlov 2003, Tsalolikhin 1995, Tsalolikhin 2001, World Flora Online 2021).

### Taxa included

**Table:** 

Rank	Scientific Name	
kingdom	Bacteria	
kingdom	Fungi	
kingdom	Plantae	
kingdom	Animalia	
phylum	Cyanobacteria	
phylum	Ascomycota	
phylum	Basidiomycota	
phylum	Charophyta	
phylum	Rhodophyta	
phylum	Marchantiophyta	
phylum	Bryophyta	
phylum	Tracheophyta	
phylum	Arthropoda	
phylum	Chordata	

## Temporal coverage

### Notes

1905 through to 2020

## Usage licence

### Usage licence

Other

### IP rights notes

This work is licensed under a Creative Commons Attribution (CC-BY) 4.0 Licence.

## Data resources

### Data package title

Occurrences of Threatened Species included in the Third Edition of the Red Data Book of the Komi Republic (Russia)

### Resource link

https://www.gbif.org/dataset/cf750a05-25f2-459b-8891-1a1fd23d7bf8

### Alternative identifiers

cf750a05-25f2-459b-8891-1a1fd23d7bf8, http://ib.komisc.ru:8088/ipt/resource?r=redbook_komi_occ

### Number of data sets

2

### Data set 1.

#### Data set name

Occurrences of Threatened Species included in the Third Edition of the Red Data Book of the Komi Republic (Russia)

#### Data format

Darwin Core

#### Number of columns

42

#### Character set

UTF8

#### Download URL

cf750a05-25f2-459b-8891-1a1fd23d7bf8, http://ib.komisc.ru:8088/ipt/resource?r=redbook_komi_occ

#### Description

The dataset contains information about 7,187 occurrences of 438 rare species and infraspecies taxa included in the third edition of the Red Data Book of the Komi Republic. Most of the occurrences’ descriptions included the following fields: 'occurrenceID', 'scientificName', 'taxonID', 'kingdom', 'phylum', 'class', 'order', 'family', 'genus', 'specificEpithet', 'infraspecificEpithet', 'scientificNameAuthorship', 'taxonRemarks', 'basisOfRecord', 'collectionCode', 'catalogNumber', 'recordedBy', 'day', 'month', 'year', 'identifiedBy', 'associatedReferences', 'decimalLatitude', 'decimalLongitude', 'geodeticDatum', 'georeferencedBy', 'coordinateUncertaintyInMetres', 'country', 'countryCode', 'stateProvince' and 'occurrenceStatus'.

**Data set 1.** 

Column label	Column description
occurrenceID	An identifier for the Occurrence.
scientificName	The full scientific name, with authorship and date information if known.
taxonID	An identifier for the set of taxon information. The data correspond to the taxonID field in the checklist dataset.
kingdom	The full scientific name of the kingdom in which the taxon is classified.
phylum	The full scientific name of the phylum in which the taxon is classified.
class	The full scientific name of the class in which the taxon is classified.
order	The full scientific name of the order in which the taxon is classified.
family	The full scientific name of the family in which the taxon is classified.
genus	The full scientific name of the genus in which the taxon is classified.
specificEpithet	The name of the first or species epithet of the scientificName.
infraspecificEpithet	The name of the lowest or terminal infraspecific epithet of the scientificName, excluding any rank designation.
scientificNameAuthorship	The authorship information for the scientificName formatted according to the conventions of the applicable nomenclaturalCode.
taxonRank	The taxonomic rank of the most specific name in the scientificName.
taxonRemarks	Rarity status and (after the symbol " | ") information about the presence of the species in the Red Book of the Russian Federation.
basisOfRecord	The specific nature of the data record.
institutionCode	The name (or acronym) in use by the institution having custody of the object(s) or information referred to in the record.
collectionCode	The name, acronym, code or initialism identifying the collection or dataset from which the record was derived.
catalogNumber	An identifier for the record within the dataset or collection.
recordedBy	A list (concatenated and separated) of names of people, groups or organisations responsible for recording the original Occurrence.
day	The integer day of the month on which the occurrence was recorded.
month	The integer month on which the occurrence was recorded.
year	The integer year on which the occurrence was recorded.
eventDate	The date-time or interval during which an Event occurred. For occurrences, this is the date-time when the event was recorded. Not suitable for a time in a geological context.
identifiedBy	A list (concatenated and separated) of names of people, groups or organisations who assigned the Taxon to the subject.
associatedReferences	A list (concatenated and separated) of identifiers (publication, bibliographic reference, global unique identifier, URI) of literature associated with the Occurrence.
decimalLatitude	The geographic latitude (in decimal degrees, using the spatial reference system given in geodeticDatum) of the geographic centre of a Location. Positive values are north of the Equator, negative values are south of it. Legal values lie between -90 and 90, inclusive.
decimalLongitude	The geographic longitude (in decimal degrees, using the spatial reference system given in geodeticDatum) of the geographic centre of a Location. Positive values are east of the Greenwich Meridian, negative values are west of it. Legal values lie between -180 and 180, inclusive.
geodeticDatum	The ellipsoid, geodetic datum or spatial reference system (SRS) upon which the geographic coordinates given in decimalLatitude and decimalLongitude are based.
georeferencedBy	A list (concatenated and separated) of names of people, groups or organisations who determined the georeference (spatial representation) for the Location.
coordinateUncertaintyInMetres	The horizontal distance (in metres) from the given decimalLatitude and decimalLongitude describing the smallest circle containing the whole of the Location.
verbatimLocality	The original textual description of the place.
country	The name of the country or major administrative unit in which the Location occurs.
countryCode	The standard code for the country in which the Location occurs.
stateProvince	The name of the next smaller administrative region than country (state, province, canton, department, region etc.) in which the Location occurs.
county	The full, unabbreviated name of the next smaller administrative region than stateProvince (county, shire, department etc.) in which the Location occurs.
locality	The specific description of the place.
habitat	A category or description of the habitat in which the Event occurred.
verbatimElevation	The original description of the elevation (altitude, usually above sea level) of the Location.
verbatimEventDate	The verbatim original representation of the date and time information for an Event.
occurrenceStatus	A statement about the presence or absence of a Taxon at a Location.
associatedTaxa	A list (concatenated and separated) of identifiers or names of taxa and the associations of this Occurrence to each of them.
associatedMedia	The URL of corresponding page image of the Third Edition of the Red Data Book of the Komi Republic.

### Data set 2.

#### Data set name

Checklist of Threatened Species included in the Third Edition of the Red Data Book of the Komi Republic (Russia)

#### Data format

Darwin Core

#### Number of columns

15

#### Character set

UTF8

#### Download URL

https://www.gbif.org/dataset/9c054c62-00e6-46e7-ab9e-421fefd8398e

#### Description

The dataset contains taxonomic information about 532 rare species and infraspecies taxa included in the third edition of the Red Data Book of the Komi Republic.

**Data set 2.** 

Column label	Column description
taxonID	An identifier for the set of taxon information. Specific to the dataset.
kingdom	The full scientific name of the kingdom in which the taxon is classified.
phylum	The full scientific name of the phylum in which the taxon is classified.
class	The full scientific name of the class in which the taxon is classified.
order	The full scientific name of the order in which the taxon is classified.
family	The full scientific name of the family in which the taxon is classified.
genus	The full scientific name of the genus in which the taxon is classified.
specificEpithet	The name of the first or species epithet of the scientificName.
infraspecificEpithet	The name of the lowest or terminal infraspecific epithet of the scientificName, excluding any rank designation.
scientificNameAuthorship	The authorship information for the scientificName formatted according to the conventions of the applicable nomenclaturalCode.
taxonRank	The taxonomic rank of the most specific name in the scientificName.
scientificName	The full scientific name, with authorship and date information, if known.
nameAccordingTo	The reference to the source in which the specific taxon concept circumscription is defined or implied - traditionally signified by the Latin "sensu" or "sec." (from secundum, meaning "according to").
taxonRemarks	Rarity status and (after the symbol " | ") information about the presence of the species in the Red Book of the Russian Federation and (after the symbol " | ") the URL of corresponding page image of the Third Edition of the Red Data Book of the Komi Republic.
vernacularName	A common or vernacular name in Russian and Komi languages, separated by " | " symbol.

## Figures and Tables

**Table 1.** Rare species, which are included in the Red Data Book of the Komi Republic (Degteva 2019), Red Data Book of the Russian Federation (Danilov-Danilyan 2001, Bardunov and Novikov 2008) and the IUCN Red List of Endangered Species (IUCN 2021)

**Species**	**Category of the rarity status**		
	**Red Data Book of the Komi Republic**	**Red Data Book of the Russian Federation**	**IUCN Red List of Endangered Species**
**PLANTS**			
**Vascular plants**			
*Anemonastrumbiarmiense* (Juz.) Holub	3*^1^	Annex	–
*Astragalusgorodkovii* Jurtz.	1	Annex	–
*Calypsobulbosa* (L.) Oakes	3	3*^1^	–
Castillejaarctica Kryl. et Serg. subsp. vorkutensis Rebr.	3	3	–
*Cotoneastercinnabarinus* Juz.	3	3	–
*Cypripediumcalceolus* L.	3	3	LC
*Dactylorhizabaltica* (Klinge) Orlova	3	3	–
*D.traunsteineri* (Saut.) Soó s.l.	3	3	–
*Epipogiumaphyllum* Sw.	2	2	–
*Isoëteslacustris* L.	3	3	–
*I.setacea* Durieu	2	2	–
*Liparisloeselii* (L.) Rich.	2	2	–
*Pseudoroegneriareflexiaristata* (Nevski) A. Lavrenko	2	Annex	–
*Rhodiolarosea* L.	3	3	–
**Bryophytes (liverworts)**			
*Cephaloziellaintegerrima* (Lindb.) Warnst.	2	2	–
*Haplomitriumhookeri* (Lyell ex Sm.) Nees	2	2	–
*Nardiabreidleri* (Limpr.) Lindb.	4	4	–
*Oleolophoziaperssonii* (H.Buch et S.W.Arnell) L.Söderstr., De Roo et Hedd.	3	3	–
*Protolophoziaelongata* (Steph.) Schljakov.	2	2	–
**Rodophyta**			
*Charastrigosa* A. Braun	3	3	–
**ANIMALS**			
**Invertebrates**			
***Crustaceans***			
*Pontastacusleptodactylus* Eschsch	1		LC
***Insects***			
*Bombusmodestus* Eversmann, 1852	2	Annex 3	DD
*B.muscorum* (Fabricius, 1775)	3	Annex 3	–
*Dytiscuslatissimus* Linnaeus, 1758	3	–	VU
*Parnassiusmnemosyne* (Linnaeus, 1758)	2	2	NT
*Parnassusphoebus* (Fabricius, 1793)	3	Annex	–
*Pyrgusandromedae* (Wallengren, 1873)	3	–	LC
*Saturniapavonia* (Linnaeus, 1758)	3	Annex 3	–
**Vertebrates**			
***Fishes***			
*Acipenserbaerii* Brandt, 1869	3	2	EN
*Cottusgobio* Linnaeus, 1758	Annex 1	2	LC
*Huchotaimen* Pallas, 1773	0	1	VU
*Salvelinusalpinus* (Linnaeus, 1758)	3	–	LC
*Stenodusleucichthys* nelma (Pallas, 1811)	1	1	LC
*Thymallusarcticus* (Pallas, 1776)	3	–	NA
***Birds***			
*Anseranser* (Linnaeus, 1758)	4	–	LC
*Ansererythropus* (Linnaeus, 1758)	2	2	VU
*Aquilachrysaetos* (Linnaeus, 1758)	3	3	LC
*A.clanga* Pallas, 1811	2	2	VU
*Botaurusstellaris* (Linnaeus, 1758)	4	–	LC
*Bubobubo* (Linnaeus, 1758)	2	2	LC
*Circusaeruginosus* (Linnaeus, 1758)	4	–	LC
*Circusmacrourus* (S.G. Gmelin, 1771)	3	2	NT
*Cygnusbewickii* Yarrell, 1830	5	2	LC
*C.cygnus* (Linnaeus, 1758)	4	–	LC
*Falcoperegrinus* Tunstall, 1771	2	2	LC
*F.rusticolus* Linnaeus, 1758	2	2	LC
*F.vespertinus* Linnaeus, 1766	3	–	NT
*Gaviaarcticaarctica* (Linnaeus, 1758)	3	2	LC
*Grusgrus* (Linnaeus, 1758)	3	–	LC
*Haematopusostraleguslongipes* Buturlin, 1910	3	3	NT
*Haliaeetusalbicilla* (Linnaeus, 1758)	3	3	LC
*Ixobrychusminutus* (Linnaeus, 1766)	4	–	LC
*Laniusexcubitor* Linnaeus, 1758	3	3	LC
*Limosalimosa* (Linnaeus, 1758)	5	–	NT
*Nycteascandiaca* (Linnaeus, 1758)	4	–	VU
*Pandionhaliaetus* (Linnaeus, 1758)	3	3	LC
*Podicepsauritus* (Linnaeus, 1758)	4	–	VU
*Rufibrentaruficollis* (Pallas, 1769)	3	3	VU
*Strixaluco* Linnaeus, 1758	3	–	LC
*S.nebulosa* J.R. Forster, 1772	3	–	LC
*S.uralensis* Pallas, 1771	3	–	LC
***Mammals***			
*Melesmeles* Linnaeus, 1758	3	–	LC
*Mustelalutreola* Linnaeus, 1761	1	Annex 3	LC
*Ochotonahyperborea* Pallas, 1811	3	–	LC
*Rangifertarandus* Linnaeus, 1758	3	–	LC (for Europe) VU (global)
**FUNGI**			
*Ganodermalucidum* (Curtis) P. Karst.	3	3	–
*Polyporusumbellatus* (Pers.) Fr.	3	3	–
*Sarcosomaglobosum* (Schmidel) Casp	2	2	–
*Sparassiscrispa* (Wulfen) Fr.	3	3	–
***Lichens***			
*Bryoriafremontii* (Tuck.) Brodo et D.Hawksw.	3	3	LC
*Leptogiumburnetiae* C.W. Dodge	3	3	–
*Leptogium rivulare (Ach.) Mont*	1	–	NT
*Lichenomphaliahudsoniana* (H.S.Jenn.) Redhead et al.	3	3	–
*Lobariapulmonaria* (L.) Hoffm.	3	2	–
*Menegazziaterebrata* (Hoffm.) A. Massal.	0	3	–
*Stereocaulondactylophyllum* Flörke	3	2	–
*Tucknerarialaureri* (Kremp.) Randlane et Thell.	3	3	–

**Table 2.** Taxonomic coverage of 'Red Data Book of the Komi Republic' and the corresponding datasets published in GBIF.

**Taxonomic group**	**Total taxa included in the Red Data Book**	**Taxa in the presented occurrence dataset**	**Taxa in GBIF data sets with occurrences on the territory of the Komi Republic published prior to presented dataset**
**Kingdom BACTERIA**	1	1	0
**Kingdom FUNGI**	150	117	33
**Kingdom PLANTS**	314	267	138
Rhodophyta	2	2	0
Charophyta	7	7	0
Bryophyte	71	28	50
Tracheophyta	233	230	88
**Kingdom ANIMALS**	68	53	36
Invertebrates	31	23	10
Vertebrates	37	30	26
Fish	5	5	0
Amphibians	1	1	1
Birds	27	20	24
Mammals	4	4	1
**Total taxa**	**532**	**438**	**207**

## Footnotes

*1 The following categories for the rarity status of species accepted in the 'Red Data Book of the Komi Republic' (Degteva 2019) and the 'Red Data Book of the Russian Federation' (Danilov-Danilyan 2001, Bardunov and Novikov 2008) were used: 0 – Probably extinct. Species (subspecies or populations) previously known to inhabit the territory of the Komi Republic whose presence in nature has not been recorded after continuous surveying efforts (for invertebrates, the last 100 years and for vertebrates, the last 50 years). 1 – Endangered species. Species (subspecies or populations) whose number of individuals has decreased to a critical level or whose habitats have decreased so much in number that they may disappear in the near future. 2 – Vulnerable species. Species (subspecies or populations) with a steadily decreasing number and/or distribution areas, which, under the further influence of factors that reduce their number, may, in a short time, fall into the category of endangered. 3 – Rare species. Species (subspecies or populations) with naturally low abundance, distributed in a limited territory (or water area) or sporadically occurring in significant territories (or water areas) that need special protection measures to ensure their survival. 4 – Indeterminate by status species. Species (subspecies or populations) that probably belong to one of the previous categories, but whose current state in nature is not well documented or that do not fully meet the criteria of all other categories, but need special protection measures. 5 – Restored and recovering species. Species (subspecies or populations) whose abundance and distribution, either due to the influence of natural causes or adopted protection measures, have begun to recover and are approaching a state where they will not need any special protection measures.
